# Evaluation of the Antioxidant Properties and Bioactivity of Koroneiki and Athinolia Olive Varieties Using In Vitro Cell-Free and Cell-Based Assays

**DOI:** 10.3390/ijms26020743

**Published:** 2025-01-16

**Authors:** Maria Gkasdrogka, Fotios Tekos, Zoi Skaperda, Periklis Vardakas, Demetrios Kouretas

**Affiliations:** Department of Biochemistry and Biotechnology, University of Thessaly, 41500 Larissa, Greece; mariagkasd@gmail.com (M.G.); fotis.tek@gmail.com (F.T.); zoiskap94@gmail.com (Z.S.); periklis_vardakas94@hotmail.com (P.V.)

**Keywords:** olives, Greek varieties, antioxidant activity, bioactivity

## Abstract

Olive oil and table olives are considered staples of the Mediterranean diet and have been associated with various health benefits. Literature reports that the final composition of the olive drupe is greatly affected by varietal and agronomic factors, each contributing to a different degree. To that end, the objective of the study was the evaluation of the contribution of different agronomic conditions applied to two Greek olive varieties (Koroneiki, Mastoidis) using a holistic approach of in vitro methods. The findings highlight the importance of the application of a combination of agronomic techniques for each variety, as marked by the differences found in the antioxidant radical-scavenging and reducing power assays. Furthermore, the results obtained from the measurement of redox biomarkers (GSH, ROS, TBARS) in cell lines (EA.hy926, HepG2, MKN45) treated with olive samples demonstrate the capacity of the samples to induce redox imbalance, either by protecting normal cells from damage, or by inducing oxidative damage in cancer cell lines, with the Athinolia samples exhibiting greater antioxidant potential at lower concentrations. This particular finding could have further applications in possible chemo-preventive approaches facilitated by antioxidant compounds of natural origins.

## 1. Introduction

The olive tree (*Olea europea* L.) is an emblematic and economically significant Mediterranean autochthonous crop [1] that contributes to the production of olive oil and table olives [2], two main staples of the Mediterranean diet [3]. The countries of the Mediterranean basin are the world’s largest producers and consumers of olive oil, with Spain, Italy, and Greece being the global leaders [4].

The Mediterranean region is characterized by seasonal fluctuations between humid and arid conditions, with extreme rainfalls and excessive heat loads; hence, the crops must be well-adapted to these environmental shifts [5]. It should be noted that the modern olive groves are genetic hybrids of wild and domesticated olive trees. The rationale behind the domestication of the olive tree was the selection of valuable agronomic traits, such as high yield and increased fruit size, as well as the increased biotic and abiotic resistance [6,7], resulting in genetic variation between modern olive trees and their ancestors [8,9].

Olive cultivation in Greece dates back to the Minoan civilization, around 3500 BC [10]. Nowadays, Greece ranks third in olive oil production globally, with Koroneiki variety (*Olea europea* var. macrocarpa alba) being one of the most prominent cultivars [11]. The primary olive-producing zones are located in Peloponnese, Crete, and the Aegean islands. The country has a distinctive geography, with olive groves found in both hilly and coastal areas [12]. It is worth mentioning that more than 170 different names of olive cultivars can be found in Greece [13], with the actual number considered to be greater than 40. Furthermore, 20 of them are grown in different places, indicating their resilience to diverse environmental conditions [14]. Amfissis, Kalamon, Koroneiki, and Athinolia varieties, in particular, are among the most extensively cultivated [15]. The Koroneiki cultivar is primarily grown in Peloponnese but also in a variety of regions throughout Greece, because of its adaptability to unfavorable climatic circumstances and its ability to thrive in arid and mountainous terrains of up to 500 m [16]. It is grown at low and medium altitudes and sufficiently humid soils, producing olive oil of exceptional quality. However, its drupe is relatively small in size, impeding the harvest procedure. Another emblematic Greek cultivar, Athinolia (*Olea europea* var. Mamillaris), contributes to the production of olive oil and table olives. It can endure in dry climates and is well-adapted to higher altitudes, allowing cultivation in areas up to 1000 m above the sea level [15,16].

Olive trees are typically grown under rainfed conditions; however, the application of an irrigation regime can have a substantial impact on yields. Irrigation is important in the production of table olives, since the desired characteristics are large, homogenous fruits with high flesh ratios, along with fruit uniformity. Therefore, irrigation systems are frequently implemented in modern olive groves [17,18]. Nevertheless, previous studies have demonstrated that irrigation can boost productivity in some cultivars, while hindering the production in others [17,19].

The olive tree and its fruits are a natural reservoir of bioactive compounds with health-promoting properties [20,21]. Olive drupes are particularly rich in phenolic substances, especially phenolic acids (hydroxybenzoic, hydroxycinnamic, caffeic, gallic), phenolic alcohols (tyrosol and hydroxytyrosol), secoiridoids (oleuropein, demethyloleuropein, ligstroside), verbascoside, anthocyanins (cyanidin and delphinidin glucosides), and flavonols (quercetin-3-rutinoside) [22,23]. The cultivar, the environmental conditions, and the agronomic practices are critical factors for the composition and concentration of these compounds in olive tree products [24], with some constituents found specifically in certain varieties [25,26]. The health benefits of olives are related to the consumption of phenols, as well as of high levels of triterpenic acids, such as maslinic and oleanolic, predominantly found in the olive skin [27,28]. These constituents have been associated with various positive effects on human health, exhibiting antioxidant, antimicrobial, and anticancer properties [29,30]. Moreover, olives contain monounsaturated and polyunsaturated fatty acids [31].

Consumer demand for high-quality food products with biofunctional properties influences farmers’ decision-making, driving them to produce higher-quality products to meet these demands. This study seeks to evaluate a large number of olive samples from the emblematic Greek varieties Koroneiki and Athinolia, two of the most prevalent and renowned cultivars in Greece known for producing high-quality olive oil. Additionally, the study examines the impact of various agronomic factors, such as location and farming practices, by employing an established holistic in vitro approach. This method enables the assessment of antioxidant potential through both antioxidant assays and studies on diverse cell lines. Consequently, our investigation aims to highlight the qualitative attributes and health benefits of these olive varieties while identifying the farming practices that contribute to the production of highly bioactive olive-derived products.

## 2. Results

### 2.1. Determination of the Antioxidant, Reducing, and Antigenotoxic Properties of Koroneiki and Athinolia Olive Samples in Cell-Free Systems

#### 2.1.1. Overall Comparison

**Figure 1:**
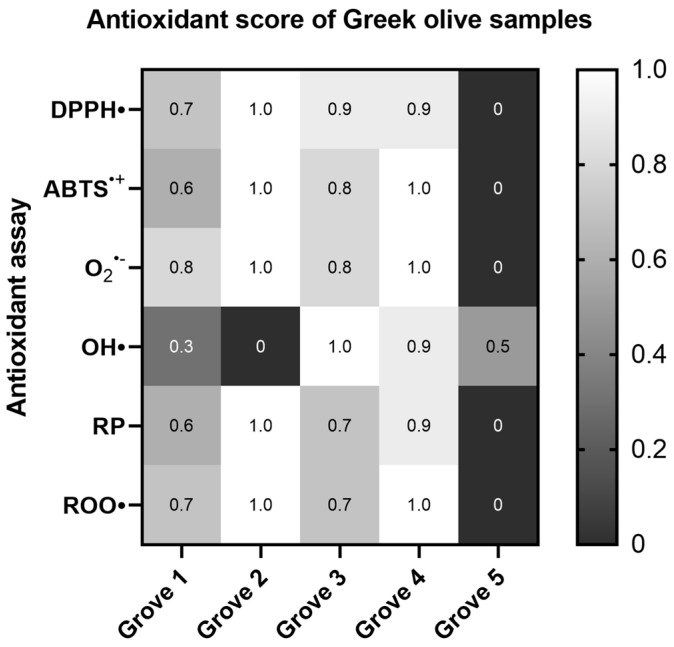
Heat map of the antioxidant score, corresponding to the antioxidant potential of Greek olive samples for the assays DPPH•, ABTS^•+^, Superoxide radical (O_2_^•−^), Hydroxyl radical (OH•), Reducing power (RP), and Peroxyl radical (ROO•)-induced plasmid DNA relaxation assay. The color scale from gray to white represents the Antioxidant score values of the normalized antioxidant capacity data, from low to high, respectively, with 0 representing the lowest antioxidant score and 1 the highest.

Our findings revealed that the olive samples had considerable antioxidant activities. However, there were significant variations between them. In general terms, the samples obtained from the olive groves of the Athinolia variety (Groves 2, 3, and 4) exhibited higher antioxidant capacity than the samples obtained from the olive groves of the Koroneiki variety (Groves 1 and 5). A more profound examination of the data revealed that the olive samples from Grove 2 (Athinolia, arid, 630 m) showed the most potent antioxidant properties, whereas the olive samples from Grove 5 (Koroneiki, irrigated, 152 m) were the least efficacious. This argument is supported by the IC50 and AU0.5 values in the majority of the antioxidant cell-free assays (Figure 1).

As regards the DPPH^•^ and O_2_^•−^ radical scavenging, and Reducing power assays, the samples labelled as Grove 2, 3, and 4 (Athinolia variety) demonstrated statistically significant differences in comparison to the sample labelled Grove 5 (Koroneiki variety). However in the ABTS^•+^ and plasmid relaxation assays, the sample labelled Grove 3 did not show statistically significant differences. Furthermore, when comparing the two different samples of the Koroneiki variety, labelled as Grove 1 and Grove 5, statistically significant differences were also observed in the O_2_^•−^ radical scavenging assay (Table S1) (Figure 1).

#### 2.1.2. Athinolia Variety

**Figure 2:**
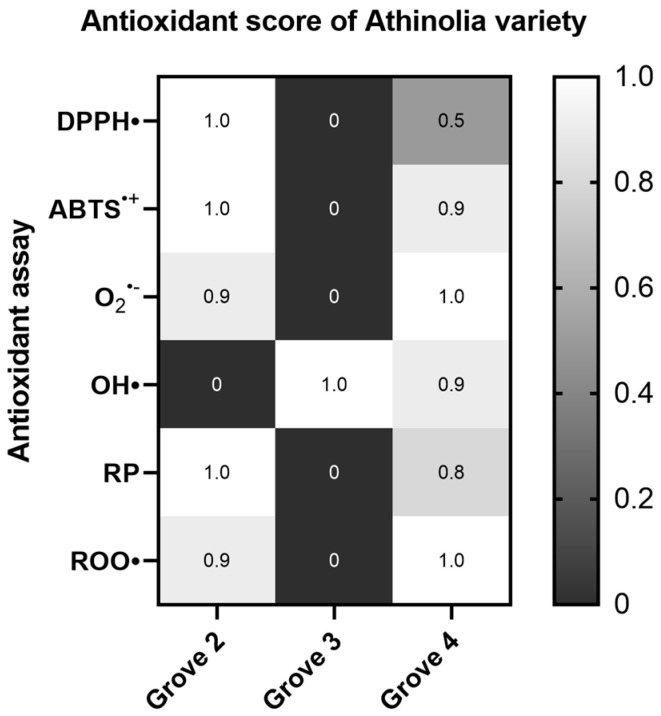
Heat map of the antioxidant score, corresponding to the antioxidant potential of Athinolia samples for the assays DPPH•, ABTS^•+^, Superoxide radical (O_2_^•−^), Hydroxyl radical (OH•), Reducing power (RP), and Peroxyl radical (ROO•)-induced plasmid DNA relaxation assay. (Grove 2: rainfed, 630 m, Grove 3: rainfed, 270 m, Grove 4: irrigated, 270 m). The color scale from gray to white represents the Antioxidant score values of the normalized antioxidant capacity data, from low to high, respectively, with 0 representing the lowest antioxidant score and 1 the highest.

When comparing the three Athinolia samples, labelled as Grove 2, 3, and 4, Grove 2 (arid conditions, 630 m) exhibited greater antioxidant capacity in all assays, with the exception of the OH^•^ radical scavenging assay. However, in the OH^•^ radical scavenging assay, the sample labelled Grove 3 exhibited greater antioxidant capacity. Statistically significant differences among the samples were observed in the OH^•^ radical and Reducing power assays (Table S2) (Figure 2).

**Figure 3:**
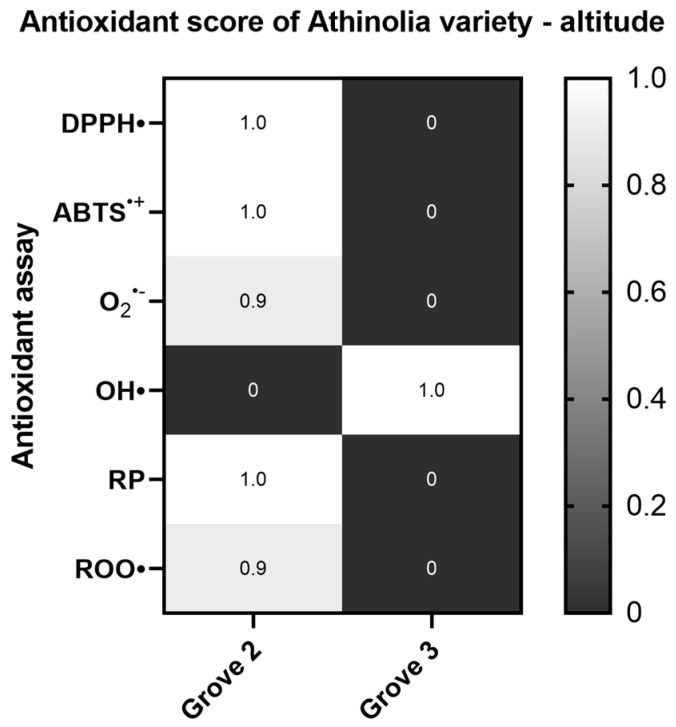
Heat map of the antioxidant score, corresponding to the antioxidant potential of Athinolia samples for the assays DPPH•, ABTS^•+^, Superoxide radical (O_2_^•−^), Hydroxyl radical (OH•), Reducing power (RP), and Peroxyl radical (ROO•)-induced plasmid DNA relaxation assay, with respect to the altitude (Grove 2: 630 m, Grove 3: 270 m). The color scale from gray to white represents the Antioxidant score values of the normalized antioxidant capacity data, from low to high, respectively, with 0 representing the lowest antioxidant score and 1 the highest.

The effect of altitude was also evaluated in the arid Athinolia samples, with the samples stemming from higher elevation being more potent. Statistically significant differences were found between the samples labelled as Grove 2 and Grove 3 in the OH^•^ radical and Reducing power assays (Table S3). However, in the OH^•^ radical scavenging assay, the sample Grove 3 was found to exhibit greater antioxidant potential (Figure 3).

**Figure 4:**
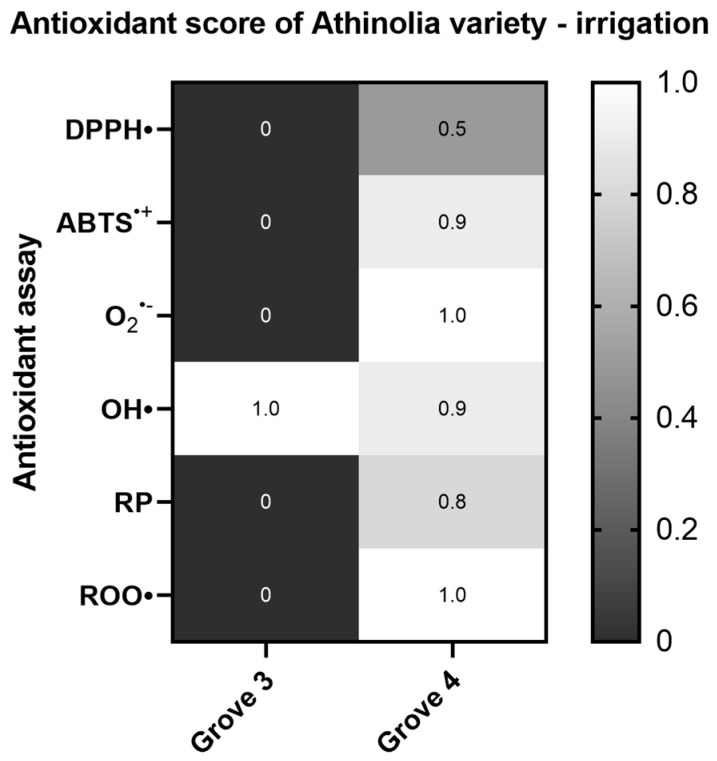
Heat map of the antioxidant score, corresponding to the antioxidant potential of Athinolia samples for the assays DPPH•, ABTS^•+^, Superoxide radical (O_2_^•−^), Hydroxyl radical (OH•), Reducing power (RP), and Peroxyl radical (ROO•)-induced plasmid DNA relaxation assay, with respect to the irrigation regime (Grove 3: rainfed, Grove 4: irrigated). The color scale from gray to white represents the Antioxidant score values of the normalized antioxidant capacity data, from low to high, respectively, with 0 representing the lowest antioxidant score and 1 the highest.

When comparing Athinolia samples deriving from the same altitude but with different irrigation strategies, no statistically significant differences were found in the majority of the assays, with the exception of the plasmid relaxation assay (Table S4), where Grove 4 (irrigated) was more potent (Figure 4).

#### 2.1.3. Koroneiki Variety

**Figure 5:**
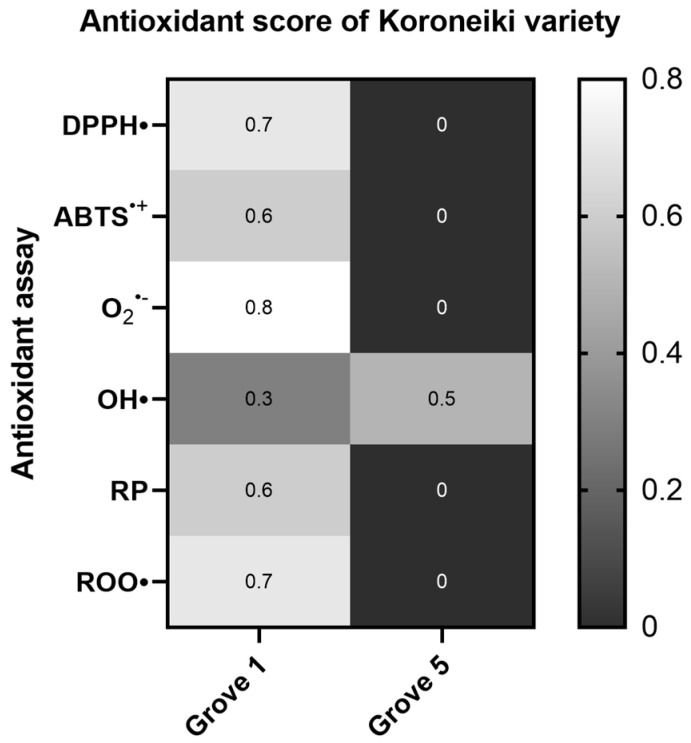
Heat map of the antioxidant score, corresponding to the antioxidant potential of Koroneiki samples for the assays DPPH•, ABTS^•+^, Superoxide radical (O_2_^•−^), Hydroxyl radical (OH•), Reducing power (RP), and Peroxyl radical (ROO•)-induced plasmid DNA relaxation assay (Grove 1: rainfed, 580 m, Grove 5: irrigated, 152 m). The color scale from gray to white represents the Antioxidant score values of the normalized antioxidant capacity data, from low to high, respectively, with 0 representing the lowest antioxidant score and 1 the highest.

Statistical analysis of the Koroneiki variety data for the Groves 1 and 5 showed similar results to those of the Athinolia variety with regards to the altitude and irrigation strategy. More specifically, in the majority of the antioxidant assays, the samples labelled as Grove 1 (arid, 580 m) exhibited greater antioxidant capacity, with statistically significant differences found in the DPPH^•^, O_2_^•−^, Reducing power, and plasmid relaxation assays (Table S5). In the OH^•^ radical scavenging assay, the same samples demonstrated higher IC50 values but the results for this assay were not statistically significant (Figure 5).

#### 2.1.4. Effect of Irrigation

**Figure 6:**
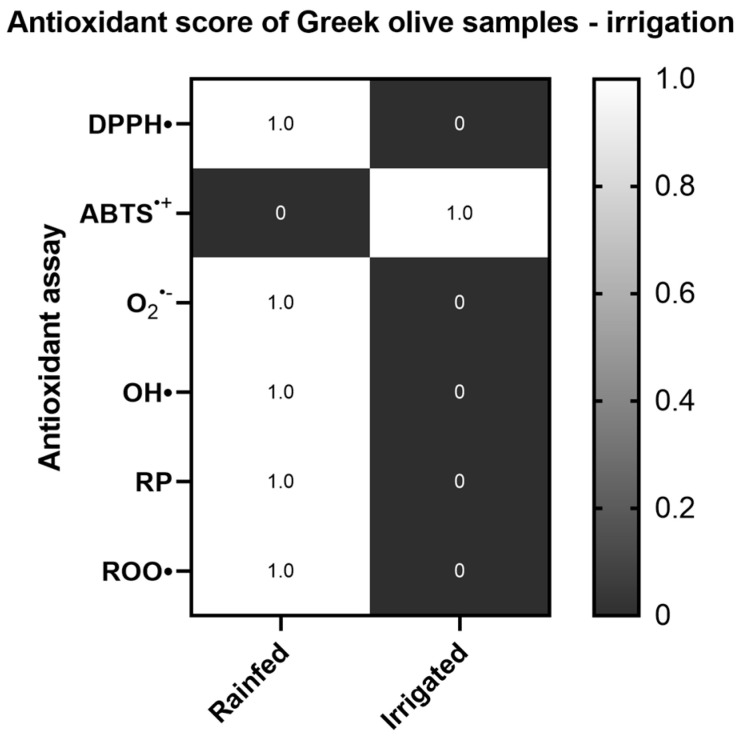
Heat map of the antioxidant score, corresponding to the antioxidant potential of Greek olive samples for the assays DPPH•, ABTS^•+^, Superoxide radical (O_2_^•−^), Hydroxyl radical (OH•), Reducing power (RP), and Peroxyl radical (ROO•)-induced plasmid DNA relaxation assay, with respect to different irrigation regimes. The color scale from grey to white represents the Antioxidant score values of the normalized antioxidant capacity data, from low to high, respectively, with 0 representing the lowest antioxidant score and 1 the highest.

Subsequently, the data of the different irrigation regimes were compared, without taking into consideration characteristics such as variety of altitude. In this case, the results showed greater antioxidant potency of the arid culture groves in most assays, with statistically significant differences found in the DPPH^•^ and Reducing power assays (Table S6) (Figure 6).

#### 2.1.5. Altitude

**Figure 7:**
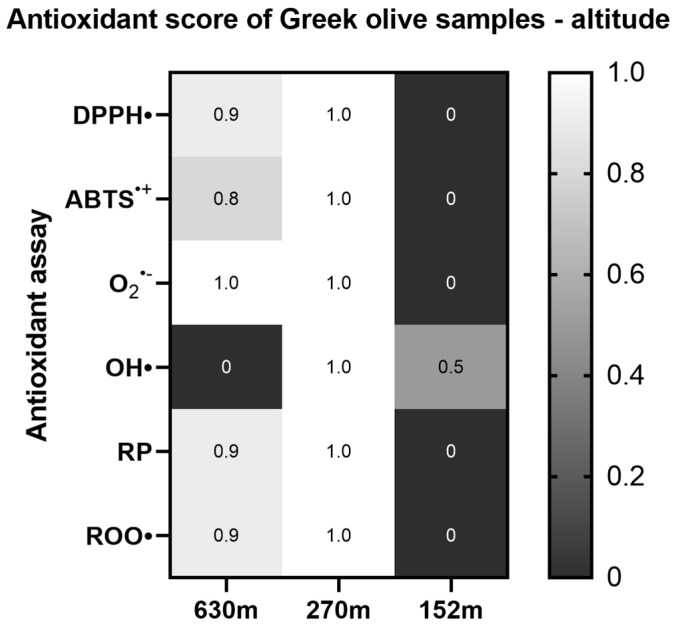
Heat map of the antioxidant score, corresponding to the antioxidant potential of Greek olive samples for the assays DPPH•, ABTS^•+^, Superoxide radical (O_2_^•−^), Hydroxyl radical (OH•), Reducing power (RP), and Peroxyl radical (ROO•)-induced plasmid DNA relaxation assay, with respect to the altitude. The color scale from gray to white represents the Antioxidant score values of the normalized antioxidant capacity data, from low to high, respectively, with 0 representing the lowest antioxidant score and 1 the highest.

The effect of the groves’ altitude was also analyzed with regard to its antioxidant ability, with the results showing that in most of the antioxidant assays the samples deriving from a higher altitude (630 m) are more potent in scavenging free radicals. Statistically significant differences were found among the samples from the two higher-elevation areas (630 m, 270 m) and the samples from the 152 m grove in the DPPH^•^, O_2_^•−^, Reducing power, and plasmid relaxation assays. Furthermore, in the ABTS^•+^ assay, statistically significant differences were observed between the groves located at 270 m and 152 m (Table S7) (Figure 7).

### 2.2. Determination of the Effects of Koroneiki and Athinolia Olive Samples on Redox Biomarkers in Cell-Based Systems

To assess the bioactivity of Koroneiki and Athinolia olive samples in cell-based systems, the aqueous phases of the olive samples collected from each grove were blended to produce five samples representative of each grove (Samples 1–5). In addition, we prepared a blend of Samples 1–5, thereafter referred to as Sample 6.

EA.hy926, HepG2, and MKN45 cells were used for investigating the bioactivity of the aforementioned samples. Prior to this, the cytotoxic threshold of each sample was determined using the XTT assay. Then, non-cytotoxic concentrations of each sample were administered in cells to evaluate their effects on redox homeostasis.

#### 2.2.1. EA.hy926 Cells

**Figure 8:**
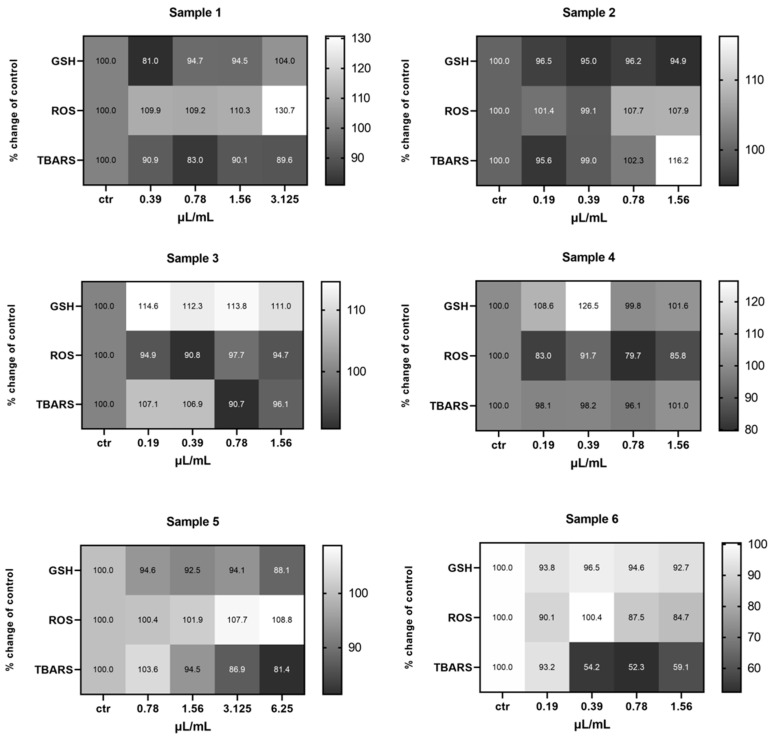
Heat map of the effects of olive samples on the levels of GSH, ROS, and TBARS on EA.hy926 cells, in comparison to the control untreated cells. The color scale from gray to white represents the percentage change of control values, from low to high, respectively, with gray representing lower values and white higher.

Treatment of the EA.hy926 cells with the test samples showed a statistically significant decrease in the GSH levels of cells treated with Sample 1 (arid, Koroneiki, 580 m), at the 0.39 μL/mL concentration compared to the control, and at 6.25 μL/mL for the cells treated with Sample 5 (irrigated, Koroneiki, 152 m). The levels of ROS were also significantly decreased in cells treated with Sample 4 (irrigated, Koroneiki, 270 m), at all of the tested concentrations, as well as in cells treated with the mixture Sample 6, at 0.78 and 1.56 μL/L. With regards to the TBARS levels, a statistically significant decrease was observed only in the cells treated with Sample 6, at the concentrations 0.39, 0.78, and 1.56 μL/mL (Tables S8–S13) (Figure 8).

#### 2.2.2. HepG2 Cells

**Figure 9:**
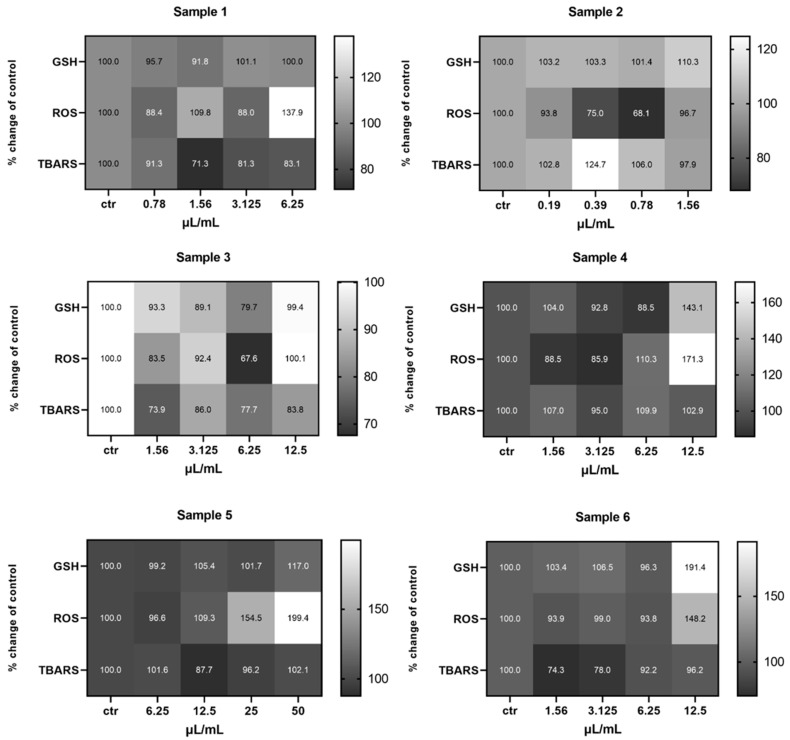
Heat map of the effects of olive samples on the levels of GSH, ROS, and TBARS on HepG2 cells, in comparison to the control untreated cells. The color scale from gray to white represents the percentage change of control values, from low to high, respectively.

Regarding the HepG2 cell line, the results showed a statistically significant increase in the GSH levels of the cells treated with Sample 4 (irrigated, Athinolia, 270 m), and the Sample 6 (mixture of all samples) at 12.5 μL/mL. As for the ROS levels, fluctuations were observed, with Sample 1 (arid, Koroneiki, 580 m) inducing a decrease at 3.125 and an increase at 6.25 μL/mL, compared to the untreated cells. Elevated levels of ROS were also observed in cells treated with Sample 5 (irrigated, Koroneiki, 152 m) at 25 and 50 μL/mL, as well as in cells treated with Sample 4 (irrigated, Athinolia, 270 m) at 12.5 μL/mL, compared to the control. Cells treated with Sample 2 (arid, Athinolia, 630 m), and Sample 3 (arid, Athinolia, 270 m) appeared to have decreased levels of ROS at 0.78 and 6.25 μL/mL, respectively. Finally, the mixture Sample 6 induced an increase of the ROS levels at 12.5 μL/mL. With respect to the TBARS levels, cells treated with Sample 1 exhibited decreased levels at 1.56 μL/mL, whereas cells treated with Sample 2 exhibited increased levels of TBARS at 0.39 μL/mL (Tables S14–S19) (Figure 9).

#### 2.2.3. MKN45 Cells

**Figure 10:**
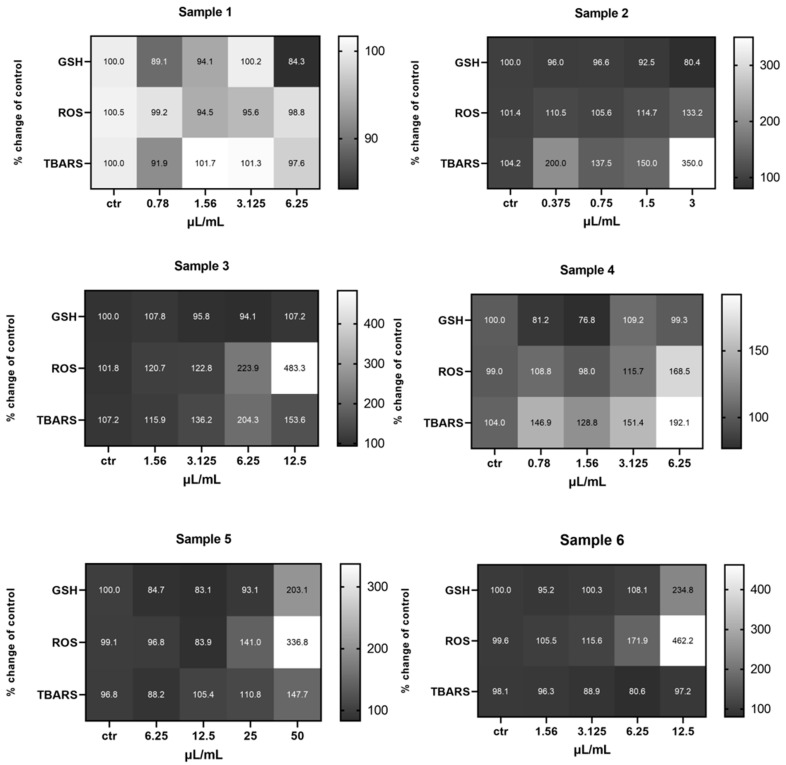
Heat map of the effects of olive samples on the levels of GSH, ROS and TBARS on MKN-45 cells, in comparison to the control untreated cells. The color scale from gray to white represents the percentage change of control values, from low to high, respectively.

With respect to the MKN45 cell line, data analysis demonstrated differences in the levels of the examined redox biomarkers on each sample. More precisely, the GSH levels of the cells treated with Sample 5 (irrigated, Koroneiki, 152 m) varied significantly, with decreased levels observed at 6.25 and 15.5 μL/mL and increased levels at 50 μL/mL. The cells treated with Sample 4 (irrigated, Athinolia, 270 m) had decreased levels of GSH at 0.78 and 1.56 μL/mL, and increased levels at 3.125 μL/mL, whereas the results for the cells treated with Sample 2 (arid, Athinolia, 630 m) and for the cells treated with the mixture Sample 6 showed a decrease at 3 μL/mL and an increase at 12.5 μL/mL, respectively. Regarding the ROS levels, in most cases an increase was observed in the treated cells. Specifically, the cells treated with Sample 3 (arid, Athinolia, 270 m) exhibited increased levels of ROS in all the test concentrations. Elevated levels of ROS were also observed in cells treated with Sample 4 at 6.25 μL/mL, in cells treated with Sample 5 at 25 and 50 μL/mL, as well as in cells treated with the mixture Sample 6, at 3.125 and 6.25 μL/mL. Finally, the TBARS levels showed variations, with decreased levels found in cells treated with Sample 1 (arid, Koroneiki, 580 m) at 0.78 μL/mL. Increased levels of TBARS were found in cells treated with Sample 2 at 3 μL/mL, in cells treated with Sample 4 at 0.78 and 3.125 μL/mL, and in cells treated with Sample 5 at 50 μL/mL (Tables S20–S25) (Figure 10).

## 3. Discussion

The present study demonstrates for the first time a holistic approach concerning the antioxidant capacity of Greek olive varieties Koroneiki and Athinolia, cultivated under different regimes, using a battery of both cell-free and in cell-based methods. Our results show that all of the tested samples, varying in certain agronomic and varietal features, are able to exert great antioxidant capacity by scavenging free radicals, as observed in the cell-free assays, as well as exhibit antigenotoxic activity. Furthermore, redox imbalance was observed in cell lines when cultured with different concentrations of the aqueous phase of Koroneiki and Athinolia olives. It is worth mentioning that variety, altitude, and irrigation factor were considered as possible variables that influence the redox biomarkers measured.

Most of the cell-free spectrophotometric antioxidant assays employed in this study showed consistent variability among the tested cultivars, but also within each cultivar, among different location and irrigation regimes. The results indicate cultivar-dependent antioxidant efficacy of the olive samples, with the Athinolia samples exhibiting greater antioxidant capacity than the samples of the Koroneiki variety, as seen by the lower IC50 and AU 0.5 values. Since different solvents and reagents can influence the antiradical activity of the samples, and in order to have an unshakeable research value proposition, it is wiser to use a variety of antioxidant assays for the determination of the total antioxidant capacity of a test sample. Furthermore, such practices will be favorable for the assessment of the compounds’ synergistic effects contained in. Therefore, the DPPH^•^, ABTS^•+^, O_2_^•−^, OH^•^, Reducing power, and plasmid relaxation assays were employed [32]. Differences in the measured IC50 and AU 0.5 values were found to be variety-related, in combination with the irrigation regime. It was found that the rainfed Athinolia sample cultivated at 630 m (Grove 2) exhibited the greatest antioxidant capacity, being able to efficiently scavenge the DPPH^•^, ABTS^•+^ radicals, as well as the OH^•^ radical, while also demonstrating great reducing capacity, and protective action against the plasmid DNA strand breaks. However, the same variety grown at 270 m under irrigated practices (Grove 4) was found to also significantly scavenge the OH^•^ radical, as well as protect plasmid DNA strand breaks. On the other hand, the Koroneiki variety samples obtained from rainfed conditions at 580 m (Grove 1), and from irrigated conditions at 152 m (Grove 5) exhibited remarkable differences in the O_2_^•−^ assay IC50 values.

Further comparison of the Athinolia variety showed notable differences due to the various altitude levels (Groves 2–4), especially among the two different rainfed samples (Grove 2 and Grove 3) as seen in the OH^•^ and Reducing power assays. However, the results differed in the two assays, with the samples from Grove 2 being less potent in the OH^•^ radical assay, while also exhibiting greater reducing capacity. What is more, when comparing the two samples obtained from 270 m (Grove 3, Grove 4), Grove 4 was able to protect the plasmid DNA strand breaks to a greater extent than Grove 3, indicating the effect of the different irrigation strategies applied at that altitude. Regarding the Koroneiki variety, it was observed that the samples acquired from Grove 1 being more potent in scavenging free radicals and exhibiting greater reducing capacity, thus demonstrating the differences in composition of the same cultivar under different agronomic techniques.

Grouping of the samples acquired from rainfed and irrigated groves revealed further impact of water availability, with samples originating from rainfed groves being more potent in all of the studied assays, with the exception of OH^•^ radical assay. The same pattern was also observed upon further comparison of the samples based on their altitude differences, with samples obtained from high and medium altitudes exhibiting greater antioxidant capacity. These analyses constitute further indication of the influence of those agronomic factors applied in modern olive groves to the composition of the final product, namely the olive drupe. Previous studies have demonstrated the capacity of olive samples to scavenge free radicals [33,34,35]. The olive tree’s response to abiotic stressors involves the accumulation of a battery of defense molecules such as phenolic compounds and antioxidant enzymes [36,37]. Its antioxidant radical scavenging capacity is largely attributed to its phenolic compounds, triterpenes, and pigments. The major phenolic classes found in the olive drupe include phenolic acids, secoiridoids, phenolic alcohols, flavonoids, and their derivatives [38,39]. It has been demonstrated that these bioactive compounds are cultivar-dependent and can be greatly affected by the aforementioned cultivation parameters [40,41,42]. Among the most extensively studied olive drupe bioactive compounds, phenolic compounds, triterpenic acids, carotenoids, as well as flavonoids and tocopherols, are worthy of mention, mainly due to their antioxidant and health-promoting capacity. Genetic factors are of great importance in determining the composition of the olive drupe [43,44], with high variability found among different cultivars [45]. Although several studies have been conducted with the aim to determine the degree of compound variation among cultivars, to this day a complete characterization of the bioactive compounds found in each cultivar has yet to come [44,46].

The olive tree is well-adapted to the semiarid conditions of the Mediterranean, where the majority of olive groves are cultivated under rainfed practices [47]. Water scarcity has been associated with elevated levels of ROS, since the absorbed light energy intended for photosynthesis is also being consumed for the generation of molecular oxygen, thus leading to the accumulation of free radicals [48]. Therefore, in order to protect their cells and compounds, plants have invested in the accumulation of antioxidant molecules involved in the antioxidant defense mechanism [49]. It has been reported that rainfed conditions may positively influence the antioxidant capacity of olives [50,51]. Agronomical factors have been shown to greatly influence the bioactive compounds found in olive drupes [44]. The majority of studies concerning olive phenolic compounds have mostly been focused on olive oil [52,53]. Phenolic compounds found in olives seem to be most influenced by the application of irrigation. However the effect of water availability appears to be affected by the type of irrigation strategy and the amount of water provided, as well as the olive variety [54]. Irrigation appears to be a critical parameter in olive cultivation, with the rainfed method still being the most-used strategy worldwide [55]. However, literature regarding the effect of irrigation applied to olive trees, with regard to polyphenol composition, seems to be contradictory, with authors reporting no statistical significant differences. Few cases suggest an increase in phenolic substances contained in irrigated olive trees [56,57] or a reduction in total phenols of olives cultivated under rainfed conditions [58]. Considering that polyphenols are secondary plant metabolites deriving from exposure to unfavourable conditions such as water shortage, plants subjected to rainfed conditions need to increase the levels of their antioxidant compounds in order to protect their cells and compounds against oxidative damage [59]. Previous studies show that increased availability of water has resulted in a reduction of the total polyphenol content of olive oils [60,61]. A study conducted in Greek olive oils obtained from the Thessaly region in Central Greece showed increased total polyphenol content in oils from rainfed trees of the Konservolia variety [62], in accordance with a different study evaluating the effects of irrigation in the Koroneiki variety in samples acquired from Crete [54]. Water restriction has also been found to positively correlated with secoiridoids levels, while other cases suggest decrease of oleuropein aglycone derivatives and tyrosol concentration [56,63,64].

Altitude is another remarkable parameter affecting the bioactivity of olives and, consequently, olive oil, with olives [65] and olive oils [66,67] originating from high and medium altitudes exhibiting higher total phenol content and increased concentration of secoiridoids and tocopherols in olive plants and olive oils obtained from higher altitude [68,69], while conversely, other findings report augmented phenolic content in olive oils from lower altitudes than those produced from higher altitudes [70,71].

Conducting such simplified in vitro systems can help identify the specific molecular interactions or pathways affected by distinct varieties of olives and olive oil cultivated under different conditions. Our results seem to be in agreement with previous literature, since the samples exhibiting greater antioxidant capacity were those originating from medium and high altitudes and cultivated under rainfed conditions. However, the results presented herein indicate strong interaction between the different cultivation practices used on each cultivar, on account of the differentiation observed among the different response of each variety under different altitude and irrigation strategy, thus suggesting the existence of different adaptation responses between the two cultivars. Taken together, these results may reflect strong varietal differences in the olive tree’s response to water availability, and therefore stress conditions.

Since obtained results indicate antioxidant potential of these Greek olive cultivars, we found worth pursuing using cell cultures to study how these samples influence cellular processes by modulating specific signaling pathways that affect metabolism. To that end, the endothelial cell line EA.hy926 was used, as a model preserving similar characteristics with the primary human endothelial cells [72], as well as two human cancer cell lines HepG2, and MKN45 with the objective to study the potential redox modulating mechanisms induced in cancer cells. Specifically, HepG2 cells is a liver cancer cell line often used in the study of redox homeostasis, since the liver is the main metabolic organ. Therefore, this cell line is employed in the studies of liver metabolism and toxicity since it retains the main characteristics of human hepatocytes. Furthermore, since olives and olive oil are products consumed by diet, it was crucial to study the effects of the test samples in the gastrointestinal tract by using the gastric cancer MKN45 cell line [73,74]. Olive cultivars with high phenol content that is mainly attributed to oleacein, oleocanthal, and oleuropein aglycon present established in vitro anti-inflammatory, antioxidant, and anticancer activity in cell cultures [14,75]. In general, many research findings show that olive oil bioactive compounds may exhibit potent anticancer effects in vitro and in vivo, in different types of cancers [76,77,78,79,80]. To our knowledge, this is the first study to demonstrate not only the protective effects of different cultivars and cultivation practices on human cells but also the dysregulation of specific pathways of the cellular antioxidant response, as indicated by the modulation of GSH, ROS, and TBARS levels. More specifically, a decrease in GSH levels after treatment of EA.hy926 cells with the Koroneiki samples (Sample 1, 5) was observed, which might be attributed to a pro-oxidant action of the samples at those concentrations. It has been reported that olive oil phenols, mainly hydroxytyrosol and oleuropein, are able to exert pro-oxidant effects due to their metal ion, mainly iron- and copper-reducing abilities, thus participating in the production of OH radicals [33,81], thus suggesting a depletion in the levels of GSH. In the endothelial EA.hy926 cells (Sample 4) of the Athinolia variety obtained from 270 m under irrigated conditions, was able to reduce the endogenous ROS levels, thus protecting the cells from the harmful effects of oxidative stress. Additionally, the samples of the Koroneiki variety (Sample 1, Sample 5) reduced the levels of GSH in the same cell line but at different concentrations, therefore indicating a difference in the composition of their bioactive compounds. Previous studies have reported the antioxidant capacity of olive oil extracts and their ability to decrease the levels of GSH in cells, therefore implying a prooxidant action of those substances [33,82]. However, in the same cell line, such an effect was not observed in cells treated with Athinolia samples. Therefore, it can be assumed that the composition of the samples, as influenced by the different varieties and agronomic factors, greatly contributes to the different antioxidant and pro-oxidant effects in cell lines. Concerning the two cancer cell lines, HepG2 and MKN45, the tested samples were able to attenuate changes in the redox status of the cells, which appears to be variety-dependent.

Several previous studies have showcased the protective effects of olive bioactive compounds when administered in various cell lines [82,83,84,85]. It has been reported that olive polyphenolic compounds exhibit antioxidant as well as pro-oxidant activities in cells [86,87]. The cytotoxic effects marked at different concentrations in the various cell lines employed in this study varied depending on the cell line, varietal differences as well as agronomic practices applied. Previous studies have demonstrated that cell proliferation may be obstructed by olive polyphenolic compounds via modulation of the endogenous ROS production [83,88], thus disturbing the redox balance of the cells. MKN45 cells treated with phenolic compounds obtained from olive fruits have exhibited reduced viability mediated through ROS reduction [89]. This finding has been utilized in cancer therapy studies, since it has been reported that cancer cells are more sensitive to polyphenol-mediated ROS manipulation compared to normal cells [83,88,89]. However, these beneficial effects in cancer cells may vary, since polyphenols can decrease or augment the basal ROS levels of cells [89]. Based on our findings, this approach could explain the differences observed in the ROS levels among the different cell lines.

Additionally, specific compounds found in the olive drupe have demonstrated redox modulating-activity in cells. Specifically, HT has been found to lead in the increase of GSH in human retinal pigment epithelial cells [82], as well as protect endothelial cells against the hazardous effects of lipid peroxidation [90] while tyrosol has the ability to mediate a decrease in ROS production [91]. Phenolic compounds have also been reported as protective substances against the damaging effects of free radicals in cell membranes [92]. Furthermore, triterpenic acids such as maslinic acid (2-α,3β-dihydroxyolean-12-en-28-oic acid) have also exhibited antioxidant activity by demonstrating protective effect against oxidative damage, as seen by the inhibition of lipid peroxidation [30]. However, this compound has also been implicated to elevated ROS levels in cells, thus promoting further cell damage and cell death via apoptosis [93,94,95]. Additionally, oleanolic acid (3β-hydroxyolean-12-en-28-oic acid), another important triterpene in olives, has exhibited protective action against oxidative stress by reinforcing the cell’s defense mechanism [96]. In cell cultures, oleanolic acid has been found to enhance the cell’s glutathione-mediated protective mechanism [97], as well as upregulating the expression of antioxidant enzymes such as GSHPx (glutathione peroxidase) and SOD (superoxide dismutase) [98,99,100].

Evidence has shown that bioactive compounds obtained from natural sources might be involved in the modulation of the nuclear factor erythroid derived 2-like (Nrf-2) pathway. Once activated, this pathway can initiate the expression of antioxidant enzymes through the translocation and binding of the Nrf2 factor in the gene promoter regions of major antioxidant genes, thus stimulating their expression [101]. Olive biophenols such as HT, tyrosol, and other compounds have been related with increased levels of GSH production in a number of cell lines by enhancing the expression of the Nrf-2 factor [86,102,103,104,105,106]. It should be noted, however, that the observed protective and antioxidant effects of polyphenolic compounds have been related to additive effects [107]. Such an effect has been observed in olive oil total extracts, which were able to exhibit greater protective action than their pure counterparts due to their compounds’ synergistic activity [108]. Most studies on the antioxidant action of olive products focus on extracts from various parts of the plant or olive oil extracts. However, a direct comparison is not feasible, as our study specifically investigates the aqueous phase of a large number of drupes sourced from a broader geographic area and influenced by diverse varietal and agronomic parameters. This is also the main competitive advantage of our study, since it utilizes, for the first time to our knowledge, the “totum” of the compounds contained in olives that have been cultivated in multiple conditions and provides the differential bioactivity levels in in vitro methodologies.

## 4. Materials and Methods

### 4.1. Chemicals and Reagents

Methanol (MeOH) was purchased from Honeywell (Honeywell, NJ, USA), 2,2-Diphenyl-1-picrylhydrazyl (DPPH^•^), 2,2′-Azinobis-(3-ethylbenzothiazoline-6-sulfonic acid) (ABTS) were purchased from Alfa Aesar (Thermo Fisher Scientific, Waltham, MA, USA), horseradish peroxidase (HRP) was purchased from SERVA (SERVA Electrophoresis GmbH, Heidelberg, Germany), and 30% H_2_O_2_ solution was purchased from Sigma-Aldrich (Merck KGaA, Darmstadt, Germany). Potassium ferricyanide [K_3_[Fe(CN)_6_] was purchased from PanReac AppliChem (ITW Reagents), iron (III) chloride from Sigma-Aldrich (Merck KGaA, Darmstadt, Germany), nitroblue tetrazolium (NBT) and nicotinamide adenine dinucleotide (NADH) were obtained from SERVA (SERVA Electrophoresis GmbH, Heidelberg, Germany), and phenazine methosulfate (PMS) and trichloroacetic acid (TCA) were purchased from Sigma-Aldrich (Merck KGaA, Darmstadt, Germany). Copper (II) chloride dihydrate (CuCl_2_) and neocuproine (Nc) were purchased from Sigma-Aldrich (Merck KGaA, Darmstadt, Germany), ammonium acetate and ethanol (EtOH) (denaturated with MEK, IPA and Bitrex^®^) 98% were obtained from Honeywell (Honeywell, NJ, USA). Agarose was purchased from SERVA (SERVA Electrophoresis GmbH, Heidelberg, Germany), phosphate-buffered saline (PBS) (0.01 M, pH = 7.4) from Gibco (Thermo Fisher Scientific, Belgium, UK), pBluescript II SK(+) was purchased from Fermentas (Waltham, MA, USA), and 2,2′-azobis(2-amidinopropane) dihydrochloride (AAPH) was obtained from Sigma-Aldrich (Merck KGaA, Darmstadt, Germany). Folin–Ciocalteu’s phenol reagent was purchased from Sigma-Aldrich (Merck KGaA, Darmstadt, Germany) and sodium carbonate anhydrous (Na_2_CO_3_) from Honeywell (Honeywell, NJ, USA). Dulbecco’s Modified Eagle’s Medium (DMEM), fetal bovine serum (FBS), antibiotic-antimycotic (100X), and trypsin were purchased from Gibco (Thermo Fisher Scientific, UK). TACS^®^ XTT cell proliferation assay kit was purchased from Roche Diagnostics (Mannheim, Germany). Intracellular GSH assay kit was obtained from Abcam (Cambridge, UK) and 2,7-dichlorofluorescein diacetate (H_2_DCFDA) was acquired from Gibco (Thermo Fisher Scientific, UK).

### 4.2. Olive Samples

The samples were acquired from the olive groves of an olive oil company located in Sparti, Peloponnese, Greece. More specifically, around 100 g of olive drupes, representative of each orchard, were collected from five distinct olive groves (Grove 1, Grove 2, Grove 3, Grove 4, Grove 5) with varying cultivars, altitudes, and irrigation regimes (Table 1). For each grove, 10 samples were obtained. The samplings were conducted on 15 and 16 December 2021, and the studied cultivars were the emblematic Greek olive varietals Koroneiki and Athinolia. Farming practices were typical of the producing area. The olive samples were collected from four different altitude zones, at 630 m, 580 m, 270 m, and 152 m. Furthermore, during the drupe growth phase (June–October) the samples from Grove 4 received irrigation volumes of 650 lt/month every 15 days and the samples from Grove 5 received irrigation volumes of 800 lt/month, four times per week.

### 4.3. Sample Preparation

A representative amount of 100 g of healthy olive drupes, without any infections or bruises, were washed and sliced, and the endocarp was removed. Then, a food processor was used to produce a homogenous olive paste. The paste was placed in tubes and centrifuged (15,000× *g*, 20 min, 4 °C). After centrifugation, three distinct phases were obtained: an oil phase on top, a paste/solid residue phase in the middle, and an aqueous phase at the bottom. The paste/solid residue phase was discarded, and the aqueous phase was transferred into 1.5 mL tubes and centrifuged anew (15,000× *g*, 10 min, 4 °C). Following centrifugation, the supernatant was transferred into new 1.5 mL tubes and stored at −20 °C until further analysis. A two-step centrifugation was employed in order to obtain only the aqueous phase of the samples. Only the aqueous phase was used for further analysis. However, due to the different agronomical conditions, the water content of the olive samples may vary, and a measurement of the water content was not performed since it does not meet the scope of the present work. Concerning the in vitro cell-based assays, we prepared a blend for each grove that included all of the grove’s samples, referred to as Samples 1 through 5. In addition, we prepared a blend of Samples 1–5, referred to as Sample 6.

### 4.4. Assessment of Antioxidant Properties In Vitro Cell-Free Systems

#### 4.4.1. DPPH^•^ Radical Scavenging Assay

The DPPH^•^ radical scavenging capacity was evaluated based on the method of Brand-Williams et al. [109] with slight modifications [82]. In brief, 50 μL of the aqueous phase diluted at various concentrations were added to 1.5 mL tubes, along with 900 μL of methanol, and 50 μL of a methanolic DPPH^•^ solution (2 mM). After incubation for 20 min in the dark at room temperature (RT), the absorbance was measured at 517 nm using a UV/Vis spectrophotometer (Hitachi U-1900 UV/Vis, Hitachi, Ltd., Tokyo, Japan). A blank sample was prepared containing 1 mL of MeOH, as well as a negative control by mixing 950 μL of MeOH with 50 μL of DPPH^•^ solution. All measurements were carried out in triplicates and at least on two separate occasions. Ascorbic acid was used as a positive control.

The radical scavenging capacity percentage (% RSC) was calculated using the following equation:

$$\%\;RSC=\frac{\left({Abs}_{control}-{Abs}_{sample}\right)}{{Abs}_{control}}\times 100$$

Abs*_control_* and Abs*_sample_* stand for the absorbance values of negative control and test sample, respectively. Furthermore, an IC50 value (half maximal inhibitory concentration) was calculated for each test sample, indicating the concentration required to scavenge 50% of the DPPH^•^ radical.

#### 4.4.2. ABTS^•+^ Radical Scavenging Assay

This assay was performed to determine the ABTS^•+^ radical scavenging capacity as described previously by Cano et al. [110], with some slight modifications [32]. Briefly, the ABTS^•+^ radicals were generated by the addition of 400 μL dH_2_O, 500 μL of an ABTS solution (1 mM), 50 μL H_2_O_2_ (30 μM), and 50 μL of a horseradish peroxidase (HRP) solution (6 μM) in 1.5 mL tubes, followed by an incubation for 45 min in the dark at RT. Then, 50 μL of the aqueous phase diluted at various concentrations were added, and the absorbance was measured at 730 nm using a UV/Vis spectrophotometer (Hitachi U-1900 UV/Vis, Hitachi, Ltd.). In each experiment, a blank was prepared without the addition of HRP and a negative control was prepared without the addition of the test sample. All experiments were carried out in triplicates and at least on two separate occasions. Ascorbic acid was used as a positive control.

The %RSC was calculated using the equation previously described for the DPPH^•^ scavenging assay, and the IC50 values were determined for each sample.

#### 4.4.3. Superoxide (O_2_^•−^) Radical Assay

The method of Gülçin et al. [111], with some minor changes [84], was performed to determine the O_2_^•−^ radical scavenging capacity. In particular, 50 μL of the aqueous phase diluted at various concentrations were added to 1.5 mL tubes and mixed with 625 μL of Tris-HCl buffer (16 mM, pH = 8.0), 125 μL of NBT solution (300 μM), and 125 μL of NADH solution (468 μM). Then, 125 μL of PMS solution (60 μM) were added to the mixture, followed by an incubation for 5 min in the dark at RT. The absorbance was measured at 560 nm using a UV/Vis spectrophotometer (Hitachi U-1900 UV/Vis, Hitachi, Ltd.). Moreover, a blank was prepared without the addition of PMS and a negative control without the addition of the test sample. All experiments were carried out in triplicates and at least on two separate occasions. Gallic acid was used as a positive control.

The %RSC percentage was calculated using the equation previously described for the DPPH^•^ scavenging assay and the IC50 values were determined.

#### 4.4.4. Hydroxyl Radical (OH^•^) Scavenging Assay

The OH^•^ scavenging capacity was assessed using the method of Osawa et al. [112], with some slight modifications [113]. In short, 75 μL of various concentrations of the aqueous phase were added to 225 μL of phosphate buffer (0.2 M, pH = 7.4), 75 μL of 2-deoxyribose solution (5 mM), 75 μL of FeSO_4_-EDTA solution (10 mM), 75 μL of H_2_O_2_ solution (10 mM), and 225 μL of dH_2_O. Following an incubation for 1 h at 37 °C, 375 μL of a 2.8% TCA solution and 375 μL of TBA solution (1% dissolved in NaOH, 50 mM) were added, the samples were incubated in a water bath at 95 °C for 20 min, and then cooled on ice for 5 min. After cooling, the samples were centrifuged (3000 rpm, 10 min, 25 °C) and the absorbance was measured at 520 nm using a UV/Vis spectrophotometer (Hitachi U-1900 UV/Vis, Hitachi, Ltd.). In each experiment, a blank sample was prepared without the addition of H_2_O_2_ and a negative control was prepared without the addition of the test sample. All experiments were carried out in triplicates and at least on two separate occasions. Caffeic acid was used as a positive control.

The %RSC percentage was calculated using the equation previously described for the DPPH^•^ scavenging assay and the IC50 values were determined.

#### 4.4.5. Reducing Power Assay

The reducing properties were evaluated using the method previously described by Yen and Duh [114], with modifications [32]. Briefly, 50 μL of the aqueous phase diluted at various concentrations, 200 μL of phosphate buffer (0.2 M, pH = 6.6), and 250 μL of potassium ferricyanide solution (1%) were mixed in 1.5 mL tubes and incubated for 20 min at 50 °C. Then, 250 μL of 10% TCA were added and the samples were centrifuged (3000 rpm, 10 min, 25 °C). Afterwards, 700 μL of the supernatant were mixed with 250 μL of dH_2_O and 50 μL of ferric chloride (0.1%), followed by incubation for 10 min in the dark at RT. The absorbance was measured at 700 nm using a UV/Vis spectrophotometer (Hitachi U-1900 UV/Vis, Hitachi, Ltd.). A blank was prepared by adding 500 μL of phosphate buffer, 250 μL of 10% TCA, 250 μL of dH_2_O, and 50 μL of ferric chloride, while the negative control contained the same reagent volumes without the test sample. All experiments were carried out in triplicates and at least on two separate occasions. Ascorbic acid was used as a positive control.

Finally, an AU0.5 value was calculated for each sample, which stands for the concentration required to achieve an absorbance value of 0.5 at 700 nm.

#### 4.4.6. Peroxyl Radical (ROO^•^)-Induced Plasmid DNA Relaxation Assay

The protective activities against ROO^•^-induced DNA strand breaks were evaluated on the basis of the method described by Paul et al. (2000) [115]. In brief, plasmid DNA (3.2 μg/mL, pBluescript SK+) (Fermentas, Waltham, MA, USA), phosphate-buffered saline (PBS), 2,2′ -azobis (2-amidinopropane hydrochloride) (AAPH) solution (95 mM in PBS), and the aqueous phase diluted at various concentrations were added to test tubes, to a final volume of 10 μL. After incubation for 45 min in the dark at 37 °C, 3 μL of loading buffer solution (bromophenol blue 0.25% + 30% glycerol) were added, following electrophoretic analysis in a 0.8% *w*/*v* agarose gel, previously stained with ethidium bromide (10 μg/mL), at 70 V for 60 min. The MultiImage Light Cabinet (Alpha Innotech, San Leandro, CA, USA) was used to expose the gel to UV, and the image was captured and analyzed using a quantification software (AlphaView software, AlphaInnotech, version 2.0.0.9, San Leandro, CA, USA). Each experiment was carried out at least twice, and a control consisting of the highest concentration of the test sample was used to check for possible strand breaks caused by the test sample. The protective effect of the sample was determined with the following equation:

$$\%\;Inhibition=\frac{S-S_{o}}{\left(S_{control}-S_{o}\right)}\times 100$$

where S*_control_* represents the % of the supercoiled plasmid DNA (negative control), S*_o_* the % of the supercoiled plasmid DNA with the AAPH radical (positive control), S the % of the supercoiled plasmid DNA with the addition of the sample and AAPH. The IC50 values were calculated as previously mentioned.

### 4.5. Assessment of Bioactivity In Vitro Cell-Based Systems

#### 4.5.1. Cell Cultures

EA.hy926 endothelial cells, HepG2 liver cancer cells, and MKN45 gastric cancer cells, cryopreserved in liquid nitrogen, were transferred into cell culture flasks 25 cm^2^ at 37 °C, 5% CO_2_, and 80–90% humidity in order to reach 70–80% confluency. According to Good Cell Culture Practice (GCCP) [116] guidelines, the cells were examined for mycoplasma contamination using the PCR method and their phenotypic characteristics were verified microscopically using an inverted microscope (OCL 251, Kern Optics). Dulbecco’s modified Eagle’s medium (DMEM) containing 4.5 g/L D-Glucose, 4 mM L-Glutamine and supplemented with 10% FBS (*v*/*v*) and 100 U/mL of penicillin and 100 U/mL of streptomycin was used for culturing EA.hy926 and HepG2 cells. Roswell Park Memorial Institute (RPMI) medium containing 2 g/L D-glucose, 2 mM L-glutamine and supplemented with 10% FBS (*v*/*v*) and 100 U/mL of penicillin and 100 U/mL of streptomycin was used for culturing MKN45 cells. The cell culture medium was changed regularly, every 2–3 days, and the cells were passaged during the log phase.

#### 4.5.2. Cell Viability Assay

Cell viability was evaluated using the XTT assay kit, as previously described [33,73]. Briefly, 1 × 10^4^ cells were seeded into a 96-well plate and cultured in their serum-supplemented medium at 37 °C, 5% CO_2_, and 80–90% humidity to reach a 70–80% confluency. Then, the cells were treated with various concentrations of the test samples, diluted in serum-free medium, for 24 h. Afterwards, 50 μL of the XTT reagent (containing 1 μL XTT activator) were added to each well, and the cells were incubated for 4 h. Following incubation, the absorbance was measured at 450 and 630 nm (reference wavelength) using a microplate reader (Bio-Tek ELx800; BioTek Instruments, Inc., Winooski, VT, USA). Untreated cells, growing in serum-free medium, were used as negative control. Additionally, the absorbance value of each test sample concentration was measured at 450 nm and subtracted from the absorbance value of cells treated with the corresponding concentration. Cell viability was calculated using the following equation:

$$Cell\;viability\;(\%\;change\;compared\;to\;control)=({Abs}_{sample}/{Abs}_{control})\times 100$$

where Abs*_control_* and Abs*_sample_* stand for the absorbance values of the negative control and test samples, respectively. All experiments were carried out in triplicates and on three separate occasions.

#### 4.5.3. Determination of Reduced Glutathione (GSH) and Reactive Oxygen Species (ROS) Levels by Flow Cytometry

The intracellular GSH and ROS levels were assessed using thiol green and 2,7-dichlorofluroscein diacetate (DCF-DA), respectively. More specifically, 3 × 10^5^ cells per well were seeded into six-well plates and cultured at 37 °C, 5% CO_2_, and 80–90% humidity to reach 70–80% confluency. Then, the cells were treated with increasing non-cytotoxic concentrations of the test samples in serum-free medium for 24 h. To determine GSH levels, the serum-free medium was discarded, and the cells were trypsinized, rinsed with PBS, and centrifuged (1500× *g*, 5 min, 4 °C). Afterwards, the supernatant was discarded, and the cell pellet was resuspended in 1 mL PBS and 5 μL thiol green and incubated for 30 min in the dark at 37 °C, with intermittent vortexing every 10 min. Following incubation, the cells were centrifuged (1200 rpm, 5 min, 4 °C), resuspended in PBS, and subjected to flow cytometric analysis. To determine ROS levels, serum-free medium was discarded, and the cells were rinsed with PBS. Then, 10 μΜ of DCF-DA were added to each well, and the cells were incubated for 30 min in the dark at 37 °C. Following incubation, the DCF-DA was removed, and the cells were trypsinized and centrifuged (1200 rpm, 5 min, 4 °C). Afterwards, the cell pellet was resuspended in PBS and centrifuged (1200 rpm, 5 min, 4 °C). Finally, the cell pellet was resuspended in PBS and subjected to flow cytometric analysis. FACSCalibur flow cytometer (Becton Dickinson, Franklin Lakes, NJ, USA) was used with excitation and emission lengths at 490/520 nm for ROS and GSH detection. Forward and right-angle light scattering, which represent cell size and internal complexity, respectively, were measured. A flow rate of ≈100 events per second was used to analyze 10,000 cells per sample. All data were analyzed using BD Cell Quest software (Becton Dickinson, Franklin Lakes, NJ, USA, version 6.0). All experiments were carried out in duplicates and at least on three separate occasions.

#### 4.5.4. Determination of Thiobarbituric Acid Reactive Substances (TBARS) Levels by Spectrophotometry

To determine TBARS levels, 7 × 10^5^ cells were seeded into cell culture flasks 25 cm^2^ and cultured in their serum-supplemented medium at 37 °C, 5% CO_2_, and 80–90% humidity to reach a 70–80% confluency. Then, the serum-supplemented medium was discarded, the cells were rinsed with PBS and treated with increasing non-cytotoxic concentrations of the test samples in serum-free medium for 24 h. Following incubation, the serum-free medium was discarded, and the cells were rinsed, trypsinized, and centrifuged (300× *g*, 5 min, 4 °C). Afterwards, the cell pellet was resuspended in PBS containing a protein-inhibitor cocktail tablet (Complete™ mini protease inhibitors, Roche Diagnostics, Mannheim, Germany), and the cells were lysed on ice using an ultrasonic processor (70% amplitude, 0.7 s pulse cycle) (UP400S, Hielscher, Teltow, Germany). Then, the cells were centrifuged (15,000× *g*, 20 min, 4 °C), and the cell lysate was collected and used to measure total protein by Bradford assay [117], as previously described [118]. Towards this purpose, a protein standard curve using serial dilutions of bovine serum albumin was generated.

TBARS levels were evaluated using the method of Keles [119], with some modifications [84]. An amount of cell lysate, containing ≈ 80 μg of total protein, was mixed with PBS and 500 μL Tris-HCl (200 mM, pH = 7.4), and 500 μL of 35% TCA were added. The samples were vortexed vigorously and incubated at RT for 10 min. Then, 1 mL of a Na_2_SO_4_ (2 M)—TBA (55 mM) solution was added, and the samples were vortexed and placed in a water bath for 45 min at 95 °C. Afterwards, the samples were transferred on ice and cooled for 5 min. After the cooling period, 1 mL of 70% TCA was added, and the samples were vortexed and centrifuged (11,200× *g*, 3 min, 25 °C). Finally, the supernatant was transferred into a cuvette, and the optical absorbance was measured at 530 nm. TBARS levels were calculated using the molar extinction coefficient of malondialdehyde (MDA), a byproduct of lipid peroxidation.

#### 4.5.5. Statistical Analysis

The Kruskal–Wallis test was performed to compare the antioxidant activities of the samples collected from different olive groves. The Data obtained from cell-free assays were analyzed using the non-parametric Kruskal–Wallis to compare the differences between cultivars, as well as using the independent samples *t*-test for comparisons between samples of the same cultivar that differed in altitude or irrigation strategies. For the cell-free data visualization, a normalization of the IC50 and AU0.5 values for each antioxidant assay was performed by using the following equation:

$$Normalized\;antioxidant\;assay=1-\frac{x-min(antioxidant\;assay)}{max\left(antioxidant\;assay\right)-min(antioxidant\;assay)}$$

All data obtained from cell-based assays were analyzed using the non-parametric Kruskal–Wallis test. Results for the in vitro cell-free assays are presented as mean ± SD, while data for the cell-based methods are presented as mean ± SEM. All analyses were performed on GraphPad Prism software, version 9.5.1 (GraphPad Software, San Diego, CA, USA). The significance level was set at *p* < 0.05.

## 5. Conclusions

In conclusion, the present study aims to highlight the degree to which different components of modern olive agriculture may contribute to the final product’s bioactivity, utilizing a holistic approach of cell-free and cell-based assays. Although the olive tree has been cultivated for millennia, there is still little knowledge on the composition of secondary metabolites and the factors influencing their concentration among Greek olive varieties. To that end, the significance of the olive cultivar was revealed as the most influential factor in terms of bioactivity, followed by the application or not of an irrigation strategy. Second, the study makes noteworthy contributions to the understanding of different adaptation mechanisms of the studied varieties, which could further contribute to the improvement of modern cultivation practices, and therefore to the production of highly bioactive products. The combination of varietal and agronomic factors demonstrated variability among the samples, as noted by their ability to modulate redox imbalance in cell lines towards human health, with the Athinolia variety exerting antioxidant and chemo-preventive effects at lower concentrations by inducing oxidative damage to cancer cells by critically raising the levels or ROS and lipid peroxidation, after administration of non-cytotoxic concentrations.

## Figures and Tables

**Table 1:** Olive grove characteristics in terms of varietal, irrigation, and altitude.

Sample	Varietal		Irrigation	Altitude (m)
	Koroneiki	Athinolia		
**Grove 1**	√		-	580
**Grove 2**		√	-	630
**Grove 3**		√	-	270
**Grove 4**		√	√	270
**Grove 5**	√		√	152

## Data Availability

Data are contained within the article and Supplementary Materials.

## Supplementary Materials

The following supporting information can be downloaded at: https://www.mdpi.com/article/10.3390/ijms26020743/s1.
