# Supplementary material for: Evaluation of the Antioxidant Properties and Bioactivity of Koroneiki and Athinolia Olive Varieties Using In Vitro Cell-Free and Cell-Based Assays
Source: Int J Mol Sci. 2025 Jan 16;26(2):743. doi: 10.3390/ijms26020743 (PMC11765908; doi:10.3390/ijms26020743)
Supplement: Supplementary file 1 [file ijms-26-00743-s001.zip › Table S1.pdf]

Table S1. Statistical analysis results for the antioxidant capacity of the test samples using one-way ANOVA for DPPH•, ABTS•+, $O_2^{\bullet-}$, OH•, Reducing power, and ROO• assays.

	Adjusted P value					
	**DPPH•**	**ABTS•+**	**$O_2^{\bullet-}$**	**OH•**	**Reducing Power**	**ROO•**
Grove 1 vs. Grove 2	0.1568	0.1818	>0.9999	>0.9999	0.1032	0.8501
Grove 1 vs. Grove 3	>0.9999	>0.9999	>0.9999	0.7671	>0.9999	>0.9999
Grove 1 vs. Grove 4	>0.9999	0.5713	>0.9999	>0.9999	>0.9999	0.5038
Grove 1 vs. Grove 5	0.1252	>0.9999	0.0079	>0.9999	0.2489	0.2634
Grove 2 vs. Grove 3	>0.9999	>0.9999	>0.9999	0.1905	0.6318	>0.9999
Grove 2 vs. Grove 4	>0.9999	>0.9999	>0.9999	0.6277	>0.9999	>0.9999
Grove 2 vs. Grove 5	<0.0001	0.0089	0.0006	>0.9999	<0.0001	0.0007
Grove 3 vs. Grove 4	>0.9999	>0.9999	>0.9999	>0.9999	>0.9999	>0.9999
Grove 3 vs. Grove 5	0.0022	0.1205	0.0311	>0.9999	0.0461	0.1173
Grove 4 vs. Grove 5	0.0004	0.0403	<0.0001	>0.9999	0.0007	0.0002
