# Supplementary material for: Evaluation of the Antioxidant Properties and Bioactivity of Koroneiki and Athinolia Olive Varieties Using In Vitro Cell-Free and Cell-Based Assays
Source: Int J Mol Sci. 2025 Jan 16;26(2):743. doi: 10.3390/ijms26020743 (PMC11765908; doi:10.3390/ijms26020743)
Supplement: Supplementary file 1 [file ijms-26-00743-s001.zip › Table S11.pdf]

Table S11. Statistical analysis results of the GSH, ROS, and TBARS levels on EA.hy926 cells, after administration of Sample 4, using one-way ANOVA for the comparison between each concentration with the control.

	**P Value**		
	**GSH**	**ROS**	**TBARS**
ctr vs. 0.19	0.6387	0.0370	0.8750
ctr vs. 0.39	0.1257	0.0384	0.8740
ctr vs. 0.78	0.9901	0.0033	0.7238
ctr vs. 1.56	0.9437	0.0114	0.9432
