# Supplementary material for: Evaluation of the Antioxidant Properties and Bioactivity of Koroneiki and Athinolia Olive Varieties Using In Vitro Cell-Free and Cell-Based Assays
Source: Int J Mol Sci. 2025 Jan 16;26(2):743. doi: 10.3390/ijms26020743 (PMC11765908; doi:10.3390/ijms26020743)
Supplement: Supplementary file 1 [file ijms-26-00743-s001.zip › Table S14.pdf]

Table S14. Statistical analysis results of the GSH, ROS, and TBARS levels on HepG2 cells, after administration of Sample 1, using one-way ANOVA for the comparison between each concentration with the control.

	**P Value**		
	**GSH**	**ROS**	**TBARS**
ctr vs. 0.78	0.7138	0.1252	0.4326
ctr vs. 1.56	0.2844	0.2060	0.0356
ctr vs. 3.125	0.9063	0.0214	0.1096
ctr vs. 6.25	0.9980	0.0372	0.1337
