# Supplementary material for: Evaluation of the Antioxidant Properties and Bioactivity of Koroneiki and Athinolia Olive Varieties Using In Vitro Cell-Free and Cell-Based Assays
Source: Int J Mol Sci. 2025 Jan 16;26(2):743. doi: 10.3390/ijms26020743 (PMC11765908; doi:10.3390/ijms26020743)
Supplement: Supplementary file 1 [file ijms-26-00743-s001.zip › Table S15.pdf]

Table S15. Statistical analysis results of the GSH, ROS, and TBARS levels on HepG2 cells, after administration of Sample 2, using one-way ANOVA for the comparison between each concentration with the control.

	**P Value**		
	**GSH**	**ROS**	**TBARS**
ctr vs. 0.19	0.5373	0.4923	0.7107
ctr vs. 0.39	0.6234	0.0618	0.0498
ctr vs. 0.78	0.8034	0.0446	0.6283
ctr vs. 1.56	0.1706	0.8027	0.8816
