# Supplementary material for: Evaluation of the Antioxidant Properties and Bioactivity of Koroneiki and Athinolia Olive Varieties Using In Vitro Cell-Free and Cell-Based Assays
Source: Int J Mol Sci. 2025 Jan 16;26(2):743. doi: 10.3390/ijms26020743 (PMC11765908; doi:10.3390/ijms26020743)
Supplement: Supplementary file 1 [file ijms-26-00743-s001.zip › Table S17.pdf]

Table S17. Statistical analysis results of the GSH, ROS, and TBARS levels on HepG2 cells, after administration of Sample 4, using one-way ANOVA for the comparison between each concentration with the control.

	**P Value**		
	**GSH**	**ROS**	**TBARS**
ctr vs. 1.56	0.7201	0.1076	0.5890
ctr vs. 3.125	0.4160	0.0849	0.4206
ctr vs. 6.25	0.2441	0.4972	0.1998
ctr vs. 12.5	0.0035	0.0003	0.9594
