# Supplementary material for: Evaluation of the Antioxidant Properties and Bioactivity of Koroneiki and Athinolia Olive Varieties Using In Vitro Cell-Free and Cell-Based Assays
Source: Int J Mol Sci. 2025 Jan 16;26(2):743. doi: 10.3390/ijms26020743 (PMC11765908; doi:10.3390/ijms26020743)
Supplement: Supplementary file 1 [file ijms-26-00743-s001.zip › Table S18.pdf]

Table S18. Statistical analysis results of the GSH, ROS, and TBARS levels on HepG2 cells, after administration of Sample 5, using one-way ANOVA for the comparison between each concentration with the control.

	**P Value**		
	**GSH**	**ROS**	**TBARS**
ctr vs. 6.25	0.9584	0.7779	0.8778
ctr vs. 12.5	0.7392	0.5727	0.2613
ctr vs. 25	0.8906	0.0322	0.7941
ctr vs. 50	0.1488	0.0270	0.9159
