# Supplementary material for: Evaluation of the Antioxidant Properties and Bioactivity of Koroneiki and Athinolia Olive Varieties Using In Vitro Cell-Free and Cell-Based Assays
Source: Int J Mol Sci. 2025 Jan 16;26(2):743. doi: 10.3390/ijms26020743 (PMC11765908; doi:10.3390/ijms26020743)
Supplement: Supplementary file 1 [file ijms-26-00743-s001.zip › Table S19.pdf]

Table S19. Statistical analysis results of the GSH, ROS, and TBARS levels on HepG2 cells, after administration of Sample 6, using one-way ANOVA for the comparison between each concentration with the control.

**P Value**			
	**GSH**	**ROS**	**TBARS**
ctr vs. 1.56	0.5247	0.4343	0.0870
ctr vs. 3.125	0.4965	0.8723	0.1538
ctr vs. 6.25	0.5230	0.5526	0.4936
ctr vs. 12.5	0.0320	0.0349	0.7373
