# Supplementary material for: Evaluation of the Antioxidant Properties and Bioactivity of Koroneiki and Athinolia Olive Varieties Using In Vitro Cell-Free and Cell-Based Assays
Source: Int J Mol Sci. 2025 Jan 16;26(2):743. doi: 10.3390/ijms26020743 (PMC11765908; doi:10.3390/ijms26020743)
Supplement: Supplementary file 1 [file ijms-26-00743-s001.zip › Table S2.pdf]

Table S2. Statistical analysis results for the antioxidant capacity of the Athinolia variety using one-way ANOVA for the DPPH•, ABTS•+, $O_2^{\bullet -}$, OH•, Reducing power, and ROO• assays.

	Adjusted P Value					
	**DPPH•**	**ABTS•+**	**$O_2^{\bullet -}$**	**OH•**	**Reducing Power**	**ROO•**
Grove 2 vs. Grove 3	0.9428	0.2107	0.4854	0.0023	0.0962	0.9919
Grove 2 vs. Grove 4	0.7727	0.9746	0.7762	0.0056	0.5332	0.4661
Grove 3 vs. Grove 4	>0.9999	0.2782	0.1810	>0.9999	>0.9999	0.4600
