# Supplementary material for: Evaluation of the Antioxidant Properties and Bioactivity of Koroneiki and Athinolia Olive Varieties Using In Vitro Cell-Free and Cell-Based Assays
Source: Int J Mol Sci. 2025 Jan 16;26(2):743. doi: 10.3390/ijms26020743 (PMC11765908; doi:10.3390/ijms26020743)
Supplement: Supplementary file 1 [file ijms-26-00743-s001.zip › Table S22.pdf]

Table S22. Statistical analysis results of the GSH, ROS, and TBARS levels on MKN-45 cells, after administration of Sample 3, using one-way ANOVA for the comparison between each concentration with the control.

	**P Value**		
	**GSH**	**ROS**	**TBARS**
ctr vs. 1.56	0.4468	0.0373	0.5848
ctr vs. 3.125	0.6319	0.0372	0.1401
ctr vs. 6.25	0.4761	0.0003	0.0637
ctr vs. 12.5	0.4743	0.0008	0.2776
