# Supplementary material for: Evaluation of the Antioxidant Properties and Bioactivity of Koroneiki and Athinolia Olive Varieties Using In Vitro Cell-Free and Cell-Based Assays
Source: Int J Mol Sci. 2025 Jan 16;26(2):743. doi: 10.3390/ijms26020743 (PMC11765908; doi:10.3390/ijms26020743)
Supplement: Supplementary file 1 [file ijms-26-00743-s001.zip › Table S24.pdf]

Table S24. Statistical analysis results of the GSH, ROS, and TBARS levels on MKN-45 cells, after administration of Sample 5, using one-way ANOVA for the comparison between each concentration with the control.

	**P Value**		
	**GSH**	**ROS**	**TBARS**
ctr vs. 6.25	0.0370	0.4738	0.3318
ctr vs. 12.5	0.0257	0.1559	0.9115
ctr vs. 25	0.5186	0.0371	0.2468
ctr vs. 50	0.0081	0.0490	0.0318
