# Supplementary material for: Evaluation of the Antioxidant Properties and Bioactivity of Koroneiki and Athinolia Olive Varieties Using In Vitro Cell-Free and Cell-Based Assays
Source: Int J Mol Sci. 2025 Jan 16;26(2):743. doi: 10.3390/ijms26020743 (PMC11765908; doi:10.3390/ijms26020743)
Supplement: Supplementary file 1 [file ijms-26-00743-s001.zip › Table S25.pdf]

Table S25. Statistical analysis results of the GSH, ROS, and TBARS levels on MKN-45 cells, after administration of Sample 6, using one-way ANOVA for the comparison between each concentration with the control.

	**P Value**		
	**GSH**	**ROS**	**TBARS**
ctr vs. 1.56	0.4650	0.2434	0.6206
ctr vs. 3.125	0.9733	0.0326	0.1978
ctr vs. 6.25	0.6986	0.0004	0.3481
ctr vs. 12.5	0.0232	0.0708	0.6219
