# Supplementary material for: Evaluation of the Antioxidant Properties and Bioactivity of Koroneiki and Athinolia Olive Varieties Using In Vitro Cell-Free and Cell-Based Assays
Source: Int J Mol Sci. 2025 Jan 16;26(2):743. doi: 10.3390/ijms26020743 (PMC11765908; doi:10.3390/ijms26020743)
Supplement: Supplementary file 1 [file ijms-26-00743-s001.zip › Table S26.pdf]

Table S26. Mean and SEM values for the GSH, ROS and TBARS levels on EA.hy926 cells after administration of Samples 1-6 for each concentration.

**GSH**			**Sample 1**					**Sample 2**					**Sample 3**		
concentration (µl/ml)	**ctr**	**0.39**	**0.78**	**1.56**	**3.125**	**ctr**	**0.19**	**0.39**	**0.78**	**1.56**	**ctr**	**0.19**	**0.39**	**0.78**	**1.56**
mean	100	80.967	94.667	94.500	104.033	100.000	96.467	95.000	96.233	94.900	99.967	114.600	112.333	113.800	110.967
sem	3.726	3.781	7.190	1.008	1.999	6.036	1.790	1.713	4.742	4.196	7.039	4.903	5.321	4.807	4.566
**ROS**															
concentration (µl/ml)	**ctr**	**0.39**	**0.78**	**1.56**	**3.125**	**ctr**	**0.19**	**0.39**	**0.78**	**1.56**	**ctr**	**0.19**	**0.39**	**0.78**	**1.56**
mean	100.000	109.867	109.167	110.267	130.700	100.000	101.400	99.133	107.733	107.933	99.967	94.867	90.833	97.667	94.700
sem	4.749	0.628	6.134	4.826	8.645	5.037	5.599	2.788	3.485	2.326	1.506	1.569	2.246	2.618	3.431
**TBARS**															
concentration (µl/ml)	**ctr**	**0.39**	**0.78**	**1.56**	**3.125**	**ctr**	**0.19**	**0.39**	**0.78**	**1.56**	**ctr**	**0.19**	**0.39**	**0.78**	**1.56**
mean	100.000	90.926	82.959	90.145	89.552	100.000	95.610	98.977	102.297	116.233	100.000	107.109	106.918	90.728	96.112
sem	5.230	6.309	4.206	3.264	6.878	8.071	8.638	13.574	5.443	6.231	1.347	5.391	3.872	5.258	5.159
**GSH**			**Sample 4**					**Sample 5**					**Sample 6**		
concentration (µl/ml)	**ctr**	**0.19**	**0.39**	**0.78**	**1.56**	**ctr**	**0.78**	**1.56**	**3.125**	**6.25**	**ctr**	**0.19**	**0.39**	**0.78**	**1.56**
mean	100.000	108.600	126.467	99.800	101.633	99.967	94.600	92.500	94.133	88.133	100.000	93.833	96.467	94.567	92.700
sem	9.025	10.469	5.685	8.426	15.014	1.242	7.398	3.132	4.250	1.157	5.033	1.137	3.538	3.445	3.492
**ROS**															
concentration (µl/ml)	**ctr**	**0.19**	**0.39**	**0.78**	**1.56**	**ctr**	**0.78**	**1.56**	**3.125**	**6.25**	**ctr**	**0.19**	**0.39**	**0.78**	**1.56**
mean	99.967	82.967	91.700	79.700	85.767	100.000	100.367	101.867	107.667	108.767	99.967	90.133	100.433	87.500	84.667
sem	1.730	3.420	1.158	0.909	1.934	3.767	3.246	2.682	2.909	1.863	2.429	0.412	1.882	1.837	1.421
**TBARS**															
concentration (µl/ml)	**ctr**	**0.19**	**0.39**	**0.78**	**1.56**	**ctr**	**0.78**	**1.56**	**3.125**	**6.25**	**ctr**	**0.19**	**0.39**	**0.78**	**1.56**
mean	100.000	98.122	98.226	96.068	101.007	100.000	103.608	94.470	86.937	81.425	100.003	93.243	54.213	52.320	59.120
sem	7.466	5.159	3.843	3.432	7.862	10.453	11.814	9.537	7.404	3.830	5.394	3.544	6.931	3.708	3.816
