# Supplementary material for: Evaluation of the Antioxidant Properties and Bioactivity of Koroneiki and Athinolia Olive Varieties Using In Vitro Cell-Free and Cell-Based Assays
Source: Int J Mol Sci. 2025 Jan 16;26(2):743. doi: 10.3390/ijms26020743 (PMC11765908; doi:10.3390/ijms26020743)
Supplement: Supplementary file 1 [file ijms-26-00743-s001.zip › Table S27.pdf]

Table S27. Mean and SEM values for the GSH. ROS and TBARS levels on HepG2 cells after administration of Samples 1-6 for each concentration.

**GSH**			**Sample 1**					**Sample 2**					**Sample 3**		
concentration (µl/ml)	**ctr**	**0.78**	**1.56**	**3.125**	**6.25**	**ctr**	**0.19**	**0.39**	**0.78**	**1.56**	**ctr**	**1.56**	**3.125**	**6.25**	**12.5**
mean	100.000	95.733	91.833	101.067	100.033	99.967	103.200	103.300	101.367	110.267	100.033	93.300	89.133	79.733	99.367
sem	1.802	8.152	4.622	6.397	9.737	3.563	0.573	3.681	2.321	3.566	8.149	4.027	5.360	7.026	5.782
**ROS**															
concentration (µl/ml)	**ctr**	**0.78**	**1.56**	**3.125**	**6.25**	**ctr**	**0.19**	**0.39**	**0.78**	**1.56**	**ctr**	**1.56**	**3.125**	**6.25**	**12.5**
mean	100.000	88.433	109.767	87.967	137.933	99.967	93.833	74.967	68.100	96.733	100.000	83.467	92.367	67.600	100.100
sem	1.391	3.913	4.414	2.056	6.567	5.938	1.728	2.121	1.050	7.847	5.216	1.944	5.649	4.491	5.354
**TBARS**															
concentration (µl/ml)	**ctr**	**0.78**	**1.56**	**3.125**	**6.25**	**ctr**	**0.19**	**0.39**	**0.78**	**1.56**	**ctr**	**1.56**	**3.125**	**6.25**	**12.5**
mean	100.000	91.282	71.333	81.311	83.134	100.000	102.805	124.718	106.009	97.919	100.000	73.910	85.953	77.740	83.823
sem	5.808	5.739	3.476	4.411	4.174	5.210	1.749	5.051	7.671	9.154	10.820	9.194	7.122	7.239	2.671
**GSH**				**Sample 4**				**Sample 5**					**Sample 6**		
concentration (µl/ml)	**ctr**	**1.56**	**3.125**	**6.25**	**12.5**	**ctr**	**6.25**	**12.5**	**25**	**50**	**ctr**	**1.56**	**3.125**	**6.25**	**12.5**
mean	100.033	103.967	92.767	88.500	143.067	100.000	99.233	105.400	101.733	117.000	99.967	103.433	106.533	96.333	191.367
sem	2.885	7.498	5.601	5.802	4.174	6.375	9.189	10.381	7.240	2.753	2.616	3.099	6.314	3.315	14.441
**ROS**															
concentration (µl/ml)	**ctr**	**1.56**	**3.125**	**6.25**	**12.5**	**ctr**	**6.25**	**12.5**	**25**	**50**	**ctr**	**1.56**	**3.125**	**6.25**	**12.5**
mean	100.000	88.533	85.867	110.267	171.300	100.000	96.633	109.300	154.467	199.433	100.000	93.900	98.967	93.800	148.233
sem	3.522	1.016	3.635	9.992	2.431	4.196	7.875	11.029	9.979	15.346	1.023	5.119	4.570	7.134	7.776
**TBARS**															
concentration (µl/ml)	**ctr**	**1.56**	**3.125**	**6.25**	**12.5**	**ctr**	**6.25**	**12.5**	**25**	**50**	**ctr**	**1.56**	**3.125**	**6.25**	**12.5**
mean	100.003	106.957	95.040	109.927	102.897	100.000	101.597	87.740	96.187	102.140	100.000	74.343	78.007	92.163	96.153
sem	5.232	4.259	11.699	4.753	6.716	6.508	4.465	1.444	8.977	13.693	7.108	5.782	7.341	4.347	4.947
