# Supplementary material for: Evaluation of the Antioxidant Properties and Bioactivity of Koroneiki and Athinolia Olive Varieties Using In Vitro Cell-Free and Cell-Based Assays
Source: Int J Mol Sci. 2025 Jan 16;26(2):743. doi: 10.3390/ijms26020743 (PMC11765908; doi:10.3390/ijms26020743)
Supplement: Supplementary file 1 [file ijms-26-00743-s001.zip › Table S28.pdf]

Table S28. Mean and SEM values for the GSH. ROS and TBARS levels on MKN-45 cells after administration of Samples 1-6 for each concentration.

**GSH**	**Sample 1**					**Sample 2**					**Sample 3**				
concentration (µl/ml)	**ctr**	**0.78**	**1.56**	**3.125**	**6.25**	**ctr**	**0.375**	**0.75**	**1.5**	**3**	**ctr**	**1.56**	**3.125**	**6.25**	**12.5**
mean	100.000	89.070	94.128	100.163	84.261	100.000	96.046	96.620	92.507	80.367	100.000	107.765	95.799	94.123	107.235
sem	0.909	5.443	12.154	7.078	5.242	3.494	5.860	3.374	1.035	3.563	0.635	7.689	6.828	6.251	7.667
**ROS**															
concentration (µl/ml)	**ctr**	**0.78**	**1.56**	**3.125**	**6.25**	**ctr**	**0.375**	**0.75**	**1.5**	**3**	**ctr**	**1.56**	**3.125**	**6.25**	**12.5**
mean	100.549	99.204	94.539	95.620	98.838	101.402	110.500	105.633	114.667	133.167	101.754	120.699	122.784	223.900	483.267
sem	1.128	1.044	3.441	3.326	0.780	1.526	4.809	6.120	9.963	14.158	4.510	3.023	8.163	9.109	25.998
**TBARS**															
concentration (µl/ml)	**ctr**	**0.78**	**1.56**	**3.125**	**6.25**	**ctr**	**0.375**	**0.75**	**1.5**	**3**	**ctr**	**1.56**	**3.125**	**6.25**	**12.5**
mean	100.000	91.867	101.700	101.333	97.567	104.167	200.000	137.500	150.000	350.000	107.246	115.942	136.232	204.348	153.623
sem	0.000	2.048	7.157	2.119	4.610	10.393	42.005	9.886	14.873	12.001	8.963	9.476	11.412	18.217	23.690
**GSH**	**Sample 4**					**Sample 5**					**Sample 6**				
concentration (µl/ml)	**ctr**	**0.78**	**1.56**	**3.125**	**6.25**	**ctr**	**6.25**	**12.5**	**25**	**50**	**ctr**	**1.56**	**3.125**	**6.25**	**12.5**
mean	100.000	81.233	76.800	109.233	99.300	100.000	84.700	83.088	93.050	203.133	100.000	95.200	100.333	108.133	234.800
sem	2.122	1.368	0.881	1.953	9.526	4.955	0.414	2.046	7.346	10.909	2.563	4.104	6.953	14.766	17.706
**ROS**															
concentration (µl/ml)	**ctr**	**0.78**	**1.56**	**3.125**	**6.25**	**ctr**	**6.25**	**12.5**	**25**	**50**	**ctr**	**1.56**	**3.125**	**6.25**	**12.5**
mean	99.033	108.750	98.000	115.700	168.500	99.100	96.840	83.850	141.033	336.800	99.567	105.467	115.633	171.867	462.200
sem	2.136	2.546	3.967	10.934	15.340	1.677	11.600	5.970	8.968	76.327	3.425	8.000	4.984	3.735	152.141
**TBARS**															
concentration (µl/ml)	**ctr**	**0.78**	**1.56**	**3.125**	**6.25**	**ctr**	**6.25**	**12.5**	**25**	**50**	**ctr**	**1.56**	**3.125**	**6.25**	**12.5**
mean	103.955	146.893	128.814	151.412	192.090	96.774	88.172	105.376	110.753	147.742	98.148	96.300	88.889	80.556	97.222
sem	8.432	14.457	20.108	4.228	21.139	8.686	5.815	8.342	10.414	15.222	5.200	17.010	9.083	6.011	6.000
