# Supplementary material for: Evaluation of the Antioxidant Properties and Bioactivity of Koroneiki and Athinolia Olive Varieties Using In Vitro Cell-Free and Cell-Based Assays
Source: Int J Mol Sci. 2025 Jan 16;26(2):743. doi: 10.3390/ijms26020743 (PMC11765908; doi:10.3390/ijms26020743)
Supplement: Supplementary file 1 [file ijms-26-00743-s001.zip › Table S4.pdf]

Table S4. Statistical analysis results for the antioxidant capacity of the Athinolia variety with respect to the irrigation regime (Grove 3: rainfed, Grove 4: irrigated), using Kruskal-Wallis for the DPPH•, ABTS•+, $O_2^{•-}$, OH•, Reducing power, and ROO• assays.

	Adjusted P Value					
	**DPPH•**	**ABTS•+**	**$O_2^{•-}$**	**OH•**	**Reducing Power**	**ROO•**
Grove 2 vs. Grove 3	0.9128	0.2043	0.0773	0.7323	0.3599	0.1544
