# Supplementary material for: Evaluation of the Antioxidant Properties and Bioactivity of Koroneiki and Athinolia Olive Varieties Using In Vitro Cell-Free and Cell-Based Assays
Source: Int J Mol Sci. 2025 Jan 16;26(2):743. doi: 10.3390/ijms26020743 (PMC11765908; doi:10.3390/ijms26020743)
Supplement: Supplementary file 1 [file ijms-26-00743-s001.zip › Table S5.pdf]

Table S5. Statistical analysis results for the antioxidant capacity of the Koroneiki variety with respect to the irrigation regime (Grove 1: rainfed, 580m, Grove 5: irrigated, 152m), using Kruskal-Wallis for the DPPH •, ABTS • +, $O_2^{•-}$, OH •, Reducing power, and ROO • assays.

	Adjusted P value					
	**DPPH•**	**ABTS•+**	**$O_2^{•-}$**	**OH•**	**Reducing Power**	**ROO•**
Grove 1 vs. Grove 5	<0.0001	0.0589	<0,0001	0.8094	0.0021	0.0019
