# Supplementary material for: Evaluation of the Antioxidant Properties and Bioactivity of Koroneiki and Athinolia Olive Varieties Using In Vitro Cell-Free and Cell-Based Assays
Source: Int J Mol Sci. 2025 Jan 16;26(2):743. doi: 10.3390/ijms26020743 (PMC11765908; doi:10.3390/ijms26020743)
Supplement: Supplementary file 1 [file ijms-26-00743-s001.zip › Table S6.pdf]

Table S6. Statistical analysis results for the antioxidant capacity of Rainfed vs. Irrigated Groves, using Kruskal-Wallis for the DPPH •, ABTS • +, $O_2^{\cdot -}$, OH •, Reducing power, and ROO • assays.

	Adjusted P value					
	**DPPH•**	**ABTS•+**	**$O_2^{\cdot -}$**	**OH•**	**Reducing Power**	**ROO•**
Rainfed vs. Irrigated	0.0021	0.1606	0.1273	0.4340	0.0007	0.0552
