# Supplementary material for: Evaluation of the Antioxidant Properties and Bioactivity of Koroneiki and Athinolia Olive Varieties Using In Vitro Cell-Free and Cell-Based Assays
Source: Int J Mol Sci. 2025 Jan 16;26(2):743. doi: 10.3390/ijms26020743 (PMC11765908; doi:10.3390/ijms26020743)
Supplement: Supplementary file 1 [file ijms-26-00743-s001.zip › Table S7.pdf]

Table S7. Statistical analysis results for the antioxidant capacity of Greek olive samples with respect to the altitude, using Kruskal-Wallis for the DPPH •, ABTS • +, $O_2^{\bullet -}$, OH •, Reducing power, and ROO • assays.

	Adjusted P value					
	**DPPH•**	**ABTS•+**	**$O_2^{\bullet -}$**	**OH•**	**Reducing Power**	**ROO•**
630m vs. 270m	>0.9999	>0.9999	>0.9999	0.0004	0.9501	0.4755
630m vs. 152m	0.0008	0.0486	<0.0001	0.5138	<0.0001	0.0003
270m vs. 152m	0.0002	0.0072	<0.0001	0.0828	<0.0001	<0.0001
