# Supplementary material for: Evaluation of the Antioxidant Properties and Bioactivity of Koroneiki and Athinolia Olive Varieties Using In Vitro Cell-Free and Cell-Based Assays
Source: Int J Mol Sci. 2025 Jan 16;26(2):743. doi: 10.3390/ijms26020743 (PMC11765908; doi:10.3390/ijms26020743)
Supplement: Supplementary file 1 [file ijms-26-00743-s001.zip › Table S8.pdf]

Table S8. Statistical analysis results of the GSH, ROS, and TBARS levels on EA.hy926 cells, after administration of Sample 1, using one-way ANOVA for the comparison between each concentration with the control.

	**P Value**		
	**GSH**	**ROS**	**TBARS**
ctr vs. 0.39	0.1431	0.681	>0.9999
ctr vs. 0.78	>0.9999	0.6819	0.1784
ctr vs. 1.56	>0.9999	0.6819	0.8050
ctr vs. 3.125	>0.9999	0.0409	0.9413
